# Perioperative Continuous Glucose Monitoring During Resection of a Giant Intrathoracic Solitary Fibrous Tumour Associated With Doege–Potter Syndrome

**DOI:** 10.1093/icvts/ivag132

**Published:** 2026-05-09

**Authors:** Kazuki Hayashi, Yusuke Kita, Ryota Nakamura, Jun Hanaoka

**Affiliations:** Department of General Thoracic Surgery, Omi Medical Center, Kusatsu, Shiga 525-8585, Japan; Department of Diabetes and Endocrinology, Omi Medical Center, Kusatsu, Shiga 525-8585, Japan; Department of General Thoracic Surgery, Omi Medical Center, Kusatsu, Shiga 525-8585, Japan; Department of Diabetes and Endocrinology, Omi Medical Center, Kusatsu, Shiga 525-8585, Japan; Department of General Thoracic Surgery, Shiga University of Medical Science, Otsu, Shiga 520-2192, Japan

**Keywords:** continuous glucose monitoring, intrathoracic solitary fibrous tumour, Doege–Potter syndrome

## Abstract

A man in his late 60s with hypertension and a history of heavy smoking presented with a large left thoracic mass. A biopsy confirmed a solitary fibrous tumour. Before his scheduled operation, he collapsed; his blood glucose level was 40 mg/dL, consistent with insulin-like growth factor-II-mediated hypoglycaemia (Doege–Potter syndrome). Continuous glucose monitoring was initiated to characterize perioperative glucose trends and support glucose management. As his consciousness and oral intake improved with supportive care and adjunctive corticosteroids, his glucose level stabilized, allowing gradual tapering of intravenous glucose. He subsequently underwent complete tumour resection via a hemi-clamshell incision. Postoperatively, his glucose level normalized immediately, and continuous glucose monitoring documented the disappearance of fluctuations by postoperative day 2. Fifteen months postoperatively, he remained recurrence-free without further hypoglycaemic episodes. Histologic analysis confirmed a solitary fibrous tumour, and Western blot analysis demonstrated high-molecular-weight insulin-like growth factor-II. This case highlights how perioperative continuous glucose monitoring can support perioperative glucose management in patients with intrathoracic solitary fibrous tumours complicated by Doege–Potter syndrome.

## INTRODUCTION

Doege–Potter syndrome (DPS) is a rare paraneoplastic complication of insulin-like growth factor-II (IGF-II)-secreting solitary fibrous tumours (SFTs), occurring in <5% of cases and typically in large intrathoracic lesions.^1^ Excess high-molecular-weight (“big”) IGF-II, characterized by a prolonged half-life and reduced binding to IGF-binding proteins, increases bioactive IGF-II and insulin-like activity, promoting peripheral glucose uptake and suppressing insulin, growth hormone, lipolysis, and ketogenesis.^1^^,^^2^ Surgical resection is the only curative therapy, although perioperative glucose control can be challenging because fluctuations are unpredictable, and intermittent testing may miss hypoglycaemia.^1^ Intraoperative continuous glucose monitoring (CGM) has been reported only once, in retroperitoneal DPS.^3^ We describe a thoracic solitary fibrous tumour with DPS in which CGM was used across the preoperative, intraoperative, and immediate postoperative phases to characterize glycaemic trends, support perioperative glucose management, and document rapid metabolic resolution after complete resection.

## CASE REPORT

**Figure 1.**
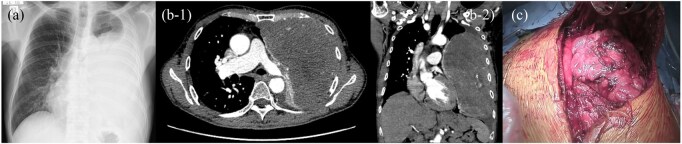
Preoperative Imaging and Intraoperative Findings. (A) Chest radiograph showing a large left thoracic mass. (B-1 and B-2) Contrast-enhanced computed tomography (axial and coronal) showing a heterogeneous mass compressing the lung with near-total atelectasis. (C) Intraoperative view through a hemi-clamshell incision showing a giant tumour adherent to pleural surfaces.

A man in his late 60s with hypertension and a history of heavy smoking presented with progressive dyspnoea and an enlarging left thoracic mass. Radiography and contrast-enhanced computed tomography showed a large heterogeneous lesion compressing the lung and mediastinum (**Figure 1a and b**). A core biopsy confirmed a pleural SFT.

While awaiting the operation, he collapsed, exhibiting incoherent speech and a glucose level of 40 mg/dL. A prior similar episode suggested recurrent hypoglycaemia. The results of laboratory studies showed suppressed insulin and C-peptide levels, a low IGF-I level (83 ng/mL), and minimal ketone elevation—findings consistent with IGF-II–mediated hypoglycaemia.^1^

**Figure 2.**
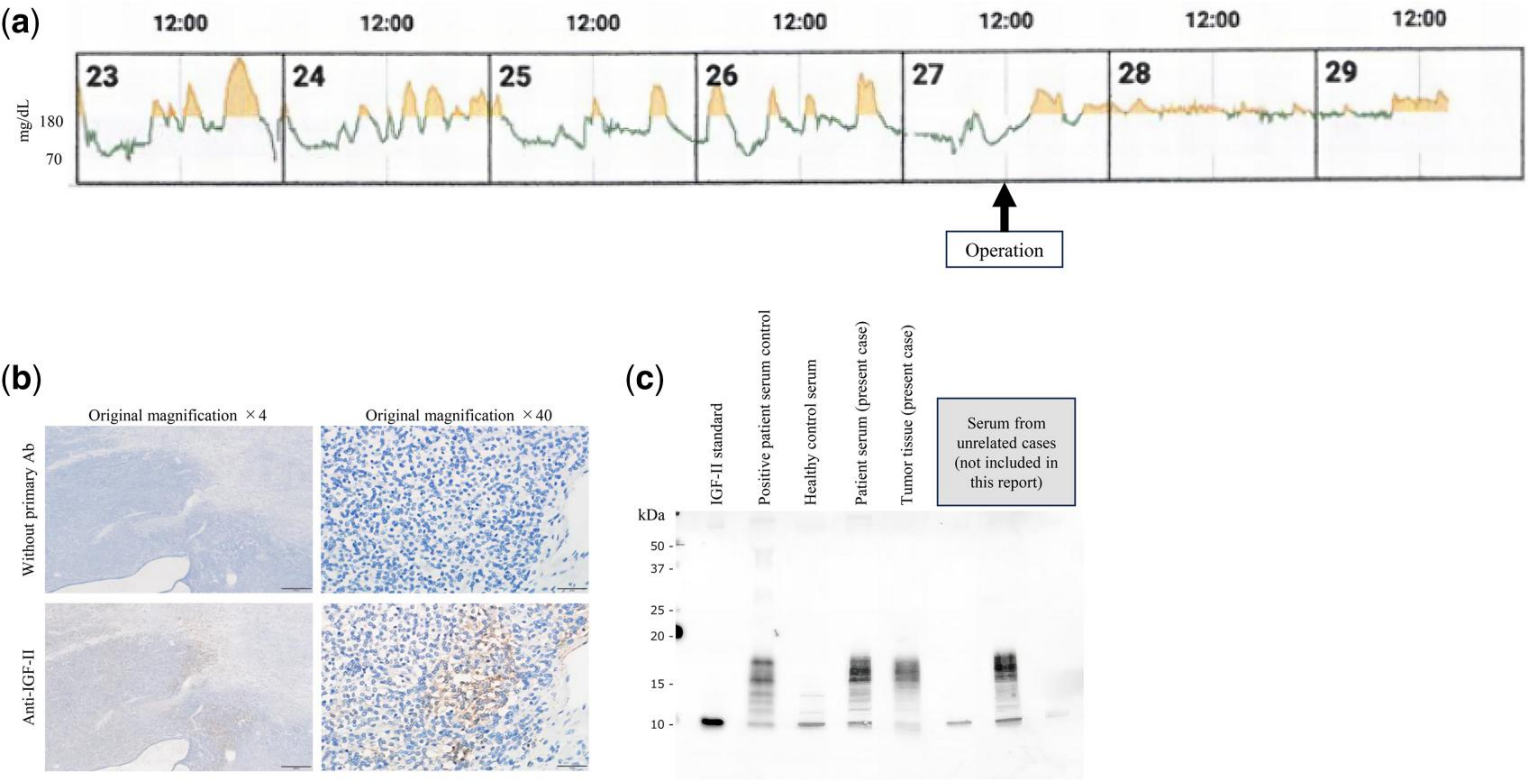
Functional and Pathological Confirmation of Insulin-like Growth Factor-II–Producing Solitary Fibrous Tumours (Doege–Potter Syndrome). (A) Continuous glucose monitoring showing preoperative glucose fluctuations with nocturnal hypoglycaemia and stable postoperative levels. (B) Insulin-like growth factor-II immunostaining showing diffuse cytoplasmic tumour positivity. (C) Western blot demonstrated high-molecular-weight insulin-like growth factor-II in preoperative serum and tumour tissue, with control sera shown for comparison. Abbreviation: IGF-II, insulin-like growth factor-II.

Despite up to 900 g/day of intravenous glucose administration, the hypoglycaemia persisted. Low-dose dexamethasone (0.5 mg/day, titrated to 2 mg/day) improved his consciousness, oral intake, and glucose stability. Continuous glucose monitoring (Dexcom G7; Dexcom Inc.) was initiated to assess daily patterns and detect nocturnal decline. Continuous glucose monitoring identified mild night-time decreases, mitigated by pre-bedtime snacks (**Figure 2a**), enabling outpatient bridging until the scheduled operation.

The patient was readmitted for resection. Given the size of the tumour and mediastinal involvement, a hemi-clamshell incision was selected for optimal exposure.^4^ The mass was densely adherent to the parietal pleura, requiring sharp dissection and limited visceral pleural resection. Complete en bloc removal was achieved (**Figure 1c**). Intraoperative CGM showed no hypoglycaemia; glucose was supplemented intermittently to maintain stable levels.

The histopathology report revealed spindle and oval cells in a collagenous stroma with focal hyalinization and myxoid changes. Mitoses were frequent (25/10 high-power fields, phosphohistone H3 positive). Immunohistochemical analysis showed STAT6, CD34, BCL-2, and CD99 positivity.^5^ IGF-II staining was diffusely positive (**Figure 2b**). Western blot analysis of serum and tumour lysates confirmed high-molecular-weight IGF-II (**Figure 2c**). According to the modified Demicco model,^5^ the tumour was high risk.

Postoperatively, the patient’s glucose level normalized immediately without supplementation. The CGM documented rapid resolution of fluctuations and was halted on postoperative day 2. The patient was discharged without complications. He remained alive 15 months after the operation without recurrence or hypoglycaemic episodes.

## DISCUSSION

In this case, the practical contribution of CGM was 3-fold: It identified nocturnal downward trends preoperatively, supported bedside adjustment of dietary intake and glucose supplementation, and objectively documented the disappearance of glycaemic variability immediately after tumour removal. THE CGM should be regarded as an adjunct to standard perioperative glucose assessment rather than a replacement for conventional blood glucose measurement. The immediate postoperative normalization of the glucose level confirmed IGF-II–mediated, tumour-dependent hypoglycaemia.

The absence of recurrent hypoglycaemia and tumour recurrence by 15 months supports the interpretation that complete resection achieved not only immediate metabolic correction but also durable disease control in this case. Complete tumour excision was essential for oncological control and metabolic cure. The hemi-clamshell incision provided wide exposure of the lung hilum and mediastinum, facilitating safe en bloc resection—consistent with reports recommending extensile approaches for large thoracic SFTs with complex adhesions.^4^

This case supports 2 practical considerations: Unexplained hypoglycaemia in the presence of a large thoracic mass should prompt consideration of DPS, and CGM may serve as a useful adjunct in perioperative glucose surveillance within multidisciplinary care.

## Data Availability

The data underlying this article are available from the corresponding author upon reasonable request.
